# The Association between Freedom of Choice and Effectiveness of Home Care Services

**DOI:** 10.5334/ijic.2448

**Published:** 2016-03-31

**Authors:** Marina Steffansson, Marjo Pulliainen, Aija Kettunen, Ismo Linnosmaa, Miikka Halonen

**Affiliations:** Research and Development Services for Social and Health Economics, Diaconia University of Applied Sciences, Pieksämäki, Finland; Centre for Health and Social Economics – CHESS, National Institute for Health and Welfare – THL, Helsinki, Finland; School of Computing, University of Eastern Finland, Joensuu, Finland

**Keywords:** freedom of choice, effectiveness, home care

## Abstract

**Objectives:**

The aim of this paper is to study home care clients’ freedom to choose their services, as well the association between the effectiveness of home care services and freedom of choice, among other factors.

**Methods:**

A structured postal survey was conducted among regular home care clients (*n* = 2096) aged 65 or older in three towns in Finland. Freedom of choice was studied based on clients’ subjective experiences. The effectiveness of the services was evaluated by means of changes in the social-care-related quality of life. Regression analyses were used to test associations.

**Results:**

As much as 62% of home care recipients reported having some choice regarding their services. Choosing meals and visiting times for the care worker were associated with better effectiveness. The basic model, which included needs and other factors expected to have an impact on quality of life, explained 15.4% of the changes in quality of life, while the extended model, which included the freedom-of-choice variables, explained 17.4%. The inclusion of freedom-of-choice variables increased the adjusted coefficient of determination by 2%. There was a significant positive association between freedom of choice and the effectiveness of public home care services.

**Conclusion:**

Freedom of choice does not exist for all clients of home care who desire it. By changing social welfare activities and structures, it is possible to show respect for clients’ opinions and to thereby improve the effectiveness of home care services.

## Introduction

Many countries aim at enhancing freedom of choice among older populations in need of social and healthcare services [1]. This theme is strongly highlighted in the recently enacted Act on Supporting the Functional Capacity of the Older Population and on Social and Health Services for Older Persons [2] in Finland. Regardless of where older people live, they wish to retain their autonomy [3]. Our objective is to investigate the association between the freedom to choose services and the quality of life of clients receiving home care services. Our focus is on home care clients in Finland. Home care refers to integrated health and social care services intended for people who need help with their daily activities [4].

Economists typically argue that clients benefit from freedom of choice. Rational consumers rank the available consumption bundles on the basis of the utility gain [5]. In order to benefit maximally from the available alternatives, consumers must have the freedom to choose between the alternatives offered to them [6,7].

Previous empirical literature on the topic has demonstrated how freedom of choice impacts the effectiveness of social services [8,9,10]. However, there is no previous research available on the association between freedom of choice and the effectiveness of home care services. The purpose of this article is to fill this gap. We used empirical data on home care clients’ freedom to choose their services to explore the associations between the effectiveness of home care services and freedom of choice, among other factors.

## Freedom of choice in care services

Freedom to choose is often linked to the concept of autonomy [9]. It is not possible to achieve a good quality of life if too many limitations are placed on a person’s autonomy. For example, the Economic and Social Research Council’s Growing Older programme has studied factors that influence the quality of life of healthy older people. Freedom of choice was among the 10 most important factors that older people listed as the most important for their quality of life [11]. However, the value of freedom of choice and autonomy in the life of older people is not uniformly understood among older people and their carers [9]. Anyone working with older people should be aware of heterogeneous preferences and needs among individuals and respect these differences in their work as a way to truly advance freedom of choice [12,13].

Duncan-Myers and Huebner [8] studied the relationship between freedom to choose and quality of life. Their results indicate a positive correlation between these two, particularly when choices are related to common tasks such as eating and freshening up [8]. The study of Vaarama, Luoma and Ylönen showed that autonomy was most respected in regard to going to bed and getting up, and the least in regard to planning daily schedules [14]. Berglund, Dunér, Blomberg & Kjellgren studied home care clients’ participation in drafting their care plans. When plans were put together, clients desired that the care worker would be the same each time because a familiar worker would make them feel safer. In addition, these clients hoped that care workers’ visiting times would be planned to suit their daily schedules [15]. Involving clients in decision-making from the beginning and trying to understand their motives has been found to improve the adaptation of services to better fit the individual needs and preferences of clients [16,17]. Receiving information about one’s choice options is significant in how freedom of choice is utilised. Older persons are exposed to a higher risk of being excluded from information because information might not be available in a form suitable for them, or it may not be sufficiently well targeted to them [18]. However, older persons are capable of expressing their opinions and providing meaningful points about their care [10].

## Importance of impairments

Individuals need care when their ability to function decreases, with services offered to compensate for this [19,20]. In basic daily activities the greatest need for help experienced by older persons was with toileting and personal hygiene, as well as with eating and drinking. In other daily activities, older persons experienced the greatest need for help with outside activities, such as moving around [14]. Some older persons see help as a relief, whereas others find it difficult to adjust to the idea that a stranger enters their lives, makes decisions on their behalf and changes their habits [21]. However, when adaptation takes place over time, they may again become able to feel pleasure regardless of the decrease in their functioning [22].

## Effectiveness of social services

Effectiveness means the desired change in the outcome, caused by an activity [23]. Decision-makers who are responsible for funding and providing the necessary social services require information about the effectiveness of services in order to make justified decisions on the allocation of scarce resources [22,24]. In the last couple of decades, parties organising social services have paid a great deal of attention to quality of life as a desired objective of services, and to the relationship between the utilisation of services and quality of life. Quality of life is based on a person’s comprehensive assessment of his or her own life and social circumstances [25].

Since the beginning of the new millennium, the well-being and quality of life of older people have been regularly studied in Finland by the National Research and Development Centre for Welfare and Health and later by its subsequent incarnation as the National Institute for Health and Welfare [7,14,26,27]. These and several other studies have shown that good health, adequate ability to function, social networks, psychological well-being and adequate livelihood are factors associated with the quality of life and well-being of older persons [6,27,28,29]. The experienced quality of life of people aged over 80 shows certain prominent features that are typical of this phase. Features decreasing quality of life include problems with functioning, dependence on help from others, experience of the appropriateness and sufficiency of help received, ability to handle tasks that require cognitive skills and a sense of insecurity [6,29,30]. Care provided at home and being able to trust that home care services are available if needed improve quality of life [7,30].

Based on economic theory and earlier empirical studies, we hypothesise that older people want to be able to choose services. The other hypotheses are that impairments and functioning as well as services that compensate for impairments are associated with the effectiveness of home care, while freedom to choose services is positively associated with effectiveness.

## Data and methods

Research data were collected during autumn 2013 and spring 2014. A structured postal survey questionnaire was sent to all regular clients aged 65 or older in the Pieksämäki and Hämeenlinna areas and Hospital District of East-Savo in Finland (*n* = 2096) who were recipients of publicly organised home care and who fulfilled the inclusion criteria. The response rate was 50.3% (*n* = 1054). Given the respondents’ advanced age and decreased capacity to function, we consider the response rate to be high. The study conformed to the ethical principles of the Declaration of Helsinki. Participants took part voluntarily and signed an informed consent. In addition, the use of client information was authorised by the social and healthcare authorities of the study areas. Given the number of questions and the fact that clients themselves completed the questionnaire, a Mini Mental Status Examination was applied as an exclusion criterion. The Mini Mental Status Examination measures cognitive impairment [31]; if a client scored under 19 points in the test, he or she was not included. If no Mini Mental Status Examination was completed, it was assumed that the client was capable of answering the questions independently. It is usual that cognitive capacity will be examined only when it appears to be decreased. The respondent’s service utilisation data were obtained from the 2013 home care client information systems administered by local authorities. In Finland, home care consists of home services provided under the Social Welfare Act [32] and home nursing provided under the Primary Health Care Act [33]. Home services are supplemented by support services, such as meals, clothing care, bathing and sauna, cleaning, help with shopping and other personal businesses, transport and errand services as well as support in social contacts [34]. The selection of support services differed slightly between the areas, and for this study we selected those support services that were common in all three study areas (Table 1).

**Table 1:** Home care services and clients in the study area.

Home services	Home nursing	Support services

– Ensure sufficient care for clients – Ensure good quality of life – Ensure autonomy for all clients through support and help with tasks that these clients are unable to manage with on their own or without the help of their relatives	– Nurse clients at home according to physician’s instructions	– Meal service – Help with shopping and other personal business – Bathing and sauna services – Safety services – Cleaning tasks
**Client:** Individuals who cannot manage their daily tasks independently or with help from their relatives or other service. Client’s care requires professional social and healthcare staff. The need for services is daily or occurs several times a week. Services are granted on the basis of individual assessments of service needs		

The freedom to choose was measured using individuals’ subjective experiences of the services they receive (see Table 2.)

**Table 2:** Questions relating to freedom of choice.

I get to choose (check where applicable)
– Care worker
– The time when my home care worker visits me (e.g. morning, daytime, evening)
– The day on which my home is cleaned
– My meals (e.g. from a menu)
I would like to influence the home care services I receive:
– Yes
– No

The effectiveness of home care was measured by studying changes in social-care-related quality of life with the adult social care outcomes toolkit (ASCOT) measure. ASCOT is a preference-weighted quality-of-life toolkit suitable for adult social care. ASCOT combines eight domains of quality of life with preferences using English preference weights [35], since there are no Finnish weights available. ASCOT has been used in a number of studies to measure social-care-related quality-of-life outcomes or effectiveness of adult social care [36,37,38]. The National Institute for Health and Care Excellence mentions ASCOT as a very rare outcome measure. The National Institute for Health and Care Excellence considers it suitable for use in measuring and valuing the effects of social care [39]. The areas and levels of quality of life in ASCOT [35] are described in Table 3.

**Table 3:** The ASCOT measure.

Domains	Levels

1) Control over daily life 2) Personal cleanliness and comfort 3) Food and drink 4) Personal safety 5) Social participation and involvement 6) Occupation 7) Accommodation cleanliness and comfort 8) Dignity	1) Ideal state: The individual’s wishes and preferences in that particular aspect of their life are fully met 2) No needs: The individual has no needs or the type of temporary trivial needs that would be expected in this area of life for someone with no impairments 3) Some needs: Some needs are distinguished from no needs by being sufficiently important or as frequently affecting an individual’s quality of life 4) High needs: High needs are distinguished from some needs by having mental or physical health implications if they are not met over a period of time. This may be because of severity or number

Version INT4 (the four-level interview tool for use with people who live in community settings) of the ASCOT toolkit was used. That version was designed initially with an interview format, and it is suitable to measure effectiveness or the outcome change due to the service use. The version measures current quality of life with services received (current social-care-related quality of life) as well as the expected quality of life without services (expected social-care-related quality of life). The difference between these two shows the change in social-care-related quality of life (social-care-related quality of life gain) [40]. In this study clients completed the questionnaire themselves. How respondents were able to cope with the number of questions posed a challenge. Especially the number and form of questions included in the ASCOT toolkit proved to be challenging for the respondents. Of all the respondents, only 517 had adequately completed the questionnaire. The missing observations were imputed by using model-based imputation methods in order to calculate the quality-of-life values. Another challenge was whether the respondents could understand properly which services the questions dealt with. Some clients also used services other than those of municipal home care. Therefore, a different inquiry form was structured for each study area, the point being to highlight to the client the services with which the questions dealt. Some ASCOT tools have been translated into Finnish using the back-translation procedure, but version INT4 is not among those. INT4 was translated for the purposes of this study using an earlier translation of version SCT4 (the four-level self-completion tool for use with people who live in community settings) that was translated according to the required procedure. Using the ASCOT INT4 interview tool as a self-completion questionnaire in addition to the translation was, as required, discussed with the ASCOT group of the University of Kent.

If older persons feel their health and/or functioning to be insufficient and have problems doing normal things for themselves, then this can influence their quality of life. Since social care aims to compensate for impairments – whether the cause is physical, mental or emotional – functioning can also influence service receivers’ experiences of care.

This is why it is recommended that, when studying quality of life, information about respondents’ health and/or ability to function should be included in the analysis [41]. To describe functioning, we used a three-level version of the EuroQol-5D (EQ-5D) measure, [42], which yields the clients’ subjective evaluations of their ability to function (Table 4).

**Table 4:** Functioning, EQ-5D measure.

Dimensions	Three-level answer options

1) Mobility (walking) 2) Self-care (washing or dressing) 3) Usual activities (work, study, housework, family or leisure activities 4) Pain/discomfort 5) Anxiety/depression	1) No problems 2) Some problems 3) Extreme problems

Our literature review showed that the quality of life of older people is associated with many factors other than simply functioning. Of these, the key variables were included in the analysis (Table 5).

**Table 5:** Characteristics of the sample included in the analyses.

Variables	

Demographic variables	Age; gender; marital status
Coping at home	Any children? Do they live at home? Does help from relatives or friends influence coping? Are the currently received home care services sufficient?
Information about home care services	Received sufficient information? More information available if needed?
Socio-economic status	Highest degree of education; subjective financial standing
Living environment	Place of living; type of housing
Utilisation of services	What home care services are in use? For how long she/he has used it?

## Methods

Data were analysed using R software. The associations between effectiveness of home care and freedom to choose and other explanatory variables were studied using multivariate regression analysis. The regression model was constructed in two phases. First we tested the assumption that the impairments and needs brought about by ageing impact effectiveness, i.e. the social-care-related quality-of-life gain. In the second phase, the model was expanded in accordance with the second assumption about the association of freedom of choice with effectiveness. When selecting the variables, the adjusted coefficient of determination was used, as it is an accepted method to test how well the model matches the data.

The models were adapted to the part of the data from which answers to the question ‘Would you like to influence the home care services you receive?’ do not suffer from missing observations. The explanatory variables in the models include only age as a continuous variable. Other explanatory variables in the data set are categorical. Variables of several categories were combined into classes. This reduces problems that might occur in the models due to the large number of explanatory variables and, in addition, improves the explanatory quality of these variables regarding effectiveness.

The residuals of the models were tested, and in both models they are relatively normally distributed. The distribution is slightly skewed to the right. Greater deviances are slightly more common in the positive direction. However, this does not influence the conclusions drawn on the basis of the coefficients and adjusted coefficients of determination in the models. Heteroscedasticity in the residuals and multicollinearity were also tested. The variance inflation factor was calculated for explanatory variables in the models, as was the generalised variance inflation factor for categorical variables. On the basis of the results of these tests, the existence of multicollinearity could be excluded.

## Results

The average age of the respondents was 84 years in both Pieksämäki and the Hospital District of East-Savo, and 85 years in Hämeenlinna (Table 6). Two-thirds of the respondents were women. Most of the respondents lived alone; about one in five lived with a spouse; only about 4% lived with their children or some other person/s. Most of the respondents lived in urban areas; one in five lived in a rural area. More than 80% of the respondents had living children. Over half of the respondents mentioned that help from friends and relatives significantly influences their coping at home. Regardless of the fact that most of the respondents had a vocational education or lower, about four in five said they felt they had sufficient income in relation to their needs. Two-thirds of the respondents considered that they have received enough information about home care services from the organiser of the service.

**Table 6:** Description of the sample.

		All *n* = 1054	Pieksämäki *n* = 233	Hämeenlinna *n* = 486	Hospital District of East-Savo *n* = 335

All (%)			22.1	46.1	31.8
Average age		84.6	84	85.4	84
Gender (%)	Male	30	30	29	31
	Female	69	70	69	69
	NA	1		2	
Marital status (%)	Unmarried	10	8	10	10
	Married	21	25	21	18
	Common law marriage	1	3	1	2
	Divorced	9	10	7	9
	Widowed	56	52	60	58
	NA	3	2	1	3
Highest education level (%)	Vocational or lower	83	88	77	85
	Tertiary	13	9	19	11
	NA	4	3	4	4
Place of living (%)	Urban area	73	73	73	77
	Rural area	18	19	18	14
	NA	9	8	9	9
Type of housing (%)	Owned	67	70	68	66
	Rented	17	16	16	18
	Rental housing for older persons	11	11	11	12
	NA	5	3	5	4
Any children? (%)	Yes	81	79	82	79
	No	16	17	15	17
	NA	3	4	3	4
Live (%)	Alone	73	72	73	74
	With someone	23	26	23	22
	NA	4	2	4	4
Received sufficient information about	Yes	64	65	60	70
services (%)	No	29	28	30	25
	NA	7	7	10	5
Help from friends and relatives influences	Significantly	56	52	60	53
coping at home (%)	Somewhat	27	29	27	27
	Not at all	12	15	8	15
	NA	5	4	5	5
Can cope when aided by the services received (%)	Yes	58	60	52	67
	No	14	12	15	13
	NA	28	28	33	20

NA, not available.

One-third of respondents had been allowed to choose the time of the care worker’s visit (Table 7). Almost as much freedom of choice was also offered for choosing the day on which to receive help with house cleaning. Freedom was weakest in regard to choosing one’s care worker. As much as two-thirds of the respondents felt that they had had the opportunity to select at least one of these items. The same number of respondents would like to influence the services they receive. However, approximately one in four respondents did not wish to influence the services received.

**Table 7:** Respondents’ experience of their possibility to choose home care services, percentage.

		All *n* = 1054	Pieksämäki *n* = 233	Hämeenlinna *n* = 486	Hospital District of East-Savo *n* = 335

Have you been able to choose?	Your care worker?	14	19	13	11
	The time of the care worker visit?	34	33	33	41
	The day to clean your home?	31	37	30	30
	Meals you desire?	20	17	22	20
	At least one of the above	63	64	61	67
Would you like to influence the home care services you receive?	Yes	62	57	64	65
	No	24	30	21	24
	NA	14	13	15	11

NA, not available.

Regarding the effectiveness of home care, the change in quality of life (social-care-related quality of life gain) was on average 0.17 (Table 8). There were differences among the areas and the difference of the mean value of the lowest and highest social-care-related quality-of-life gain was significant (Kruskal–Wallis *p* < 0.05).

**Table 8:** Change in quality of life in the study areas.

Area	*N*	Average	Median	Std. error of mean

Pieksämäki	112	0.18	0.14	0.018
Hämeenlinna	216	0.15	0.10	0.013
Hospital District of East-Savo	189	0.20	0.16	0.015
Total	517	0.17	0.12	0.009

## Regression analyses

The first basic model (Table 9) included functioning and key variables found in earlier research to be associated with the quality of life of older people. After testing, a total of 15 variables were selected out of the 31 originally in the model. The adjusted coefficient of determination of the model with these variables is 15.4%.

**Table 9:** The basic model tested the assumption that the needs brought about by ageing impact quality of life.

	Estimate	Std. error	*t* value	*Pr*(>|*t*|)

(Intercept)	–0.148	0.087	–1.695	0.09
Age	0.002	0.001	2.46	0.014*
Tertiary education	–0.027	0.017	–1.566	0.118
Rental housing for older persons	0.026	0.022	1.222	0.224
Yes, children	–0.039	0.019	–2.039	0.042*
Live with someone	–0.019	0.016	–1.182	0.238
Receive sufficient information about home care services	0.016	0.016	1.005	0.315
Help by friends or relatives impacts coping at home, slightly or not at all	0.027	0.014	1.967	0.05*
Can cope when aided by the services received	0.057	0.017	3.304	0.001**
Mobility, extreme problems	–0.026	0.02	–1.264	0.206
Self-care, some problems	0.056	0.016	3.46	0.001**
Self-care, extreme problems	0.128	0.028	4.508	0.000***
Usual activities, some problems or extreme problems	0.032	0.017	1.928	0.054
Home service, under 12 months	0.041	0.02	2.066	0.039*
Home service, over 12 months	0.079	0.016	4.844	0.000***
Meal service, 4 months or over	0.053	0.014	3.62	0.000***
Multiple R-squared: 0.1682	Adjusted R-squared: 0.1543			

Signif. Codes. *p* < 0.1, *p* < 0.05, **p < 0.01, ***p < 0.001.

Nine of the examined variables were significantly related to the effectiveness of home care. Using home services for over 12 months showed the strongest association, and using meal services for 4 months or over showed a significant association with the effectiveness of home care. Impairments and need for help showed a strong association as well. One of the five dimensions in the EQ-5D instrument, self-care (ability to wash or dress) was strongly associated with the effectiveness of home care, and problems with normal activities were also selected for the model. The domains of pain/discomfort and anxiety/depression from the EQ-5D were dropped from the model during the testing procedure. In addition to the above variables, the effectiveness of home care was associated with age and with the situation in which friends and relatives help only slightly or not at all. A significant negative association with the effectiveness of services occurred when a respondent has children.

The first model was extended by adding freedom-of-choice variables. After testing, a total of 20 variables were selected for the extended model (Table 10) containing all five freedom-of-choice variables. The inclusion of freedom-of-choice variables increased the adjusted coefficient of determination by 2%, and the new model explained 17.4% of the change in quality of life.

**Table 10:** The extended model tested the assumption that the needs brought about by ageing and freedom of choice would impact quality of life.

	Estimate	Std. error	*t* value	*Pr*(>|*t*|)

(Intercept)	–0.105	0.085	–1.2343	0.217
Would you like to influence the services you receive?	–0.017	0.015	–1.1724	0.241
Have been able to choose the care worker	0.017	0.019	0.9012	0.368
Have been able to choose the day of the care worker’s visit	0.029	0.015	1.9973	0.046*
Have been able to choose the day for house cleaning	0.033	0.014	2.4273	0.015*
Have been able to choose the desired meal	0.045	0.016	2.7606	0.006**
Age	0.002	0.001	2.0104	0.045*
Tertiary education	–0.037	0.017	–2.1516	0.032*
Rental housing for older persons	0.031	0.021	1.4638	0.144
Yes, children	–0.039	0.019	–2.0828	0.038*
Live with someone	–0.02	0.016	–1.2419	0.215
Receive sufficient information about home care services	0.004	0.016	0.2677	0.789
Help by friends or relatives impacts coping at home, slightly or not at all	0.023	0.014	1.6862	0.092
Can cope when aided by the services received	0.048	0.017	2.7873	0.005**
Mobility, extreme problems	–0.023	0.02	–1.1556	0.248
Self-care, some problems	0.051	0.016	3.2358	0.001**
Self-care, extreme problems	0.12	0.029	4.1715	0.000***
Usual activities, some problems or extreme problems	0.026	0.017	1.5689	0.117
Home service, under 12 months	0.04	0.02	2.0128	0.044*
Home service, over 12 months	0.075	0.017	4.5185	0.000***
Meal service, 4 months or over	0.052	0.014	3.6432	0.000***
Multiple R-squared: 0.1916	Adjusted R-squared: 0.1735			

Signif. codes. *p* < 0.1, *p < 0.05, **p < 0.01, ***p < 0.001.

Of the five freedom-of-choice variables, three were significantly associated with the effectiveness of home care. The relationship is clearest in the case of the possibility to choose meals to one’s liking. The other two that showed significant associations are the possibility to choose the time of the care worker’s visit and the possibility to choose the day for housecleaning. Regarding other variables, the picture described by the extended model is quite similar to the first model, with one exception. The extended model had an additional significant negative association: home care is found to be less effective if the respondent has higher education.

## Discussion

The purpose of this paper was to study home care clients’ freedom to choose their services, as well as the associations between the effectiveness of home care and freedom of choice. Our working hypothesis was that older people want to choose their services. The other hypothesis was that impairments and functioning as well as services compensating for impairments are associated with the effectiveness of home care. We expected that freedom to choose is also associated with effectiveness. The main results of the study supported these hypotheses.

According to the postal survey data from 1054 older recipients of municipal home care in three areas in Finland, more than half reported that they would like to influence the services they received. Nevertheless, only about a third had been allowed to choose the time of the care worker’s visit or the cleaning day, and only a fifth the meal of their choice. According to our findings, home care services seem to contribute positively to the respondents’ social-care-related quality of life, indicating the effectiveness of the services. There were differences between the study areas, but explanations for these differences were not examined in this study.

Multivariate regression analyses showed that both the service use and impairments were associated with the effectiveness of home care. In addition, freedom of choice showed a positive association with the effectiveness of home care, as expected: each variable measuring choice was associated with the effectiveness of home care. The regression models were constructed in two phases, with both models being significant. The models were capable of explaining the effectiveness of home care rather well. The adjusted coefficients of determination in the models can be considered as reasonable for a study like this. Our results are similar to the findings obtained in other studies [6,7,29].

A more striking finding was that almost a quarter of the respondents did not wish to influence the services they received. The respondents’ functional ability and cognitive skills may have deteriorated such that they did not want or they did not have the capability to choose. They may also be unaware of the fact that they are entitled to choose or at least to express their opinion [18,43] or of what the service supply contains. Therefore, information intended for these clients should be communicated to them in different ways in order to account for differences between them [10]. This result emphasises how important the role of the person assessing the service need is to the client’s quality of life.

The ASCOT measure used in this study is limited in certain ways. First of all, preference weights are not available for the Finnish population. However, the results ASCOT yielded are similar to those acquired in England. According to results of earlier studies, social services influence the different components of quality of life in different ways [20,37]. The second limitation is associated with the method of using ASCOT. Although version INT4 was created for interviewing, such that the contents of the questions could be clarified for respondents when needed, they were here used as part of a postal survey. Only about half of the respondents answered adequately enough to enable an evaluation of the service.

In addition, the cross-sectional data used in this study does not allow us to estimate the causal effect of freedom of choice on the effectiveness of service use. Alternative methods, such as for example the instrument variables method or a panel data set with a time variation, would allow a better assessment of the effect of freedom of choice on the effectiveness of home care use.

Our results suggest that freedom of choice is related to the effectiveness of services. If there is a causal relationship between freedom of choice and effectiveness, our main finding is important in considering the planning of the future social services system. One of the goals of current social policy is to find ways to help older persons cope in their own homes for as long as possible. If the effectiveness of home care services is to be improved, actions must be taken and structures must be strengthened to support clients’ freedom of choice regarding their services. When service plans are drafted for home care clients, the focus is on their service needs, but it is also possible to improve the effectiveness of home care by observing clients’ own wishes.

## Conclusion

The results of this study show that freedom of choice is positively associated with the effectiveness of home care services. However, freedom of choice is not realised for all home care clients. Much remains to be done to improve clients’ quality of life and the effectiveness of public home care services. It is important to alter actions and structures in the provision of social welfare so that the voice of older persons would be better heard. Our findings are important in situations where the number of ageing people in home care is increasing, and pressures on society to manage the situation are growing.

## Competing Interests

The authors declare that they have no competing interests.
